# Replaced gastroduodenal artery with continuation as accessory left hepatic artery: a rare anatomical variant

**DOI:** 10.1186/s42155-018-0032-2

**Published:** 2018-11-30

**Authors:** Jesse Chen, Amit Ramjit, Noor Ahmad

**Affiliations:** Department of Interventional Radiology, Staten Island University Hospital, 475 Seaview Ave, Staten Island, NY 10305 USA

**Keywords:** Hepatic artery, Replaced GDA, Accessory left hepatic artery

## Abstract

**Background:**

Presented here is a rare case of a gastroduodenal artery (GDA) replaced to the superior mesenteric artery with continuation into an accessory left hepatic artery. To the authors’ knowledge, this rare anomaly is not described previously. Replaced GDA is reported, however without continuation into an accessory LHA.

**Case presentation:**

A 74-year-old male with recurrent peptic ulcer disease presented with acute onset melena. This anomaly was discovered during abdominal angiography performed for treatment of a hemorrhagic duodenal ulcer. Initial celiac and common hepatic artery (CHA) subselection and angiography demonstrated no branch vessels. Angiography via the superior mesenteric artery demonstrated free extravasation into the duodenum and distal perfusion of the left hepatic lobe.

**Conclusions:**

This is a rare variant of hepatic arterial anatomy. Importantly, this variant challenges a previously described anatomical model that attempts to explain observed CHA variation.

## Background

Variant hepatic arterial anatomy is not uncommon, with only 60% of patients having the classical arterial anatomy (Covey et al. 2002). Successful completion of interventional radiology procedures in this area can depend on knowledge of the breadth of variation of hepatic anatomy. We report a case not included in previously published large series where a gastroduodenal artery (GDA) replaced to the superior mesenteric artery (SMA) gives rise to an *accessory* left hepatic artery (LHA). This variant challenges a previously described anatomic model for summarizing observed common hepatic artery (CHA) variation. Identified during treatment of a bleeding peptic ulcer, complex variants such as this may have important ramifications for other procedures in interventional radiology including radio- and chemo-embolization.

## Case presentation

**Fig. 1:**
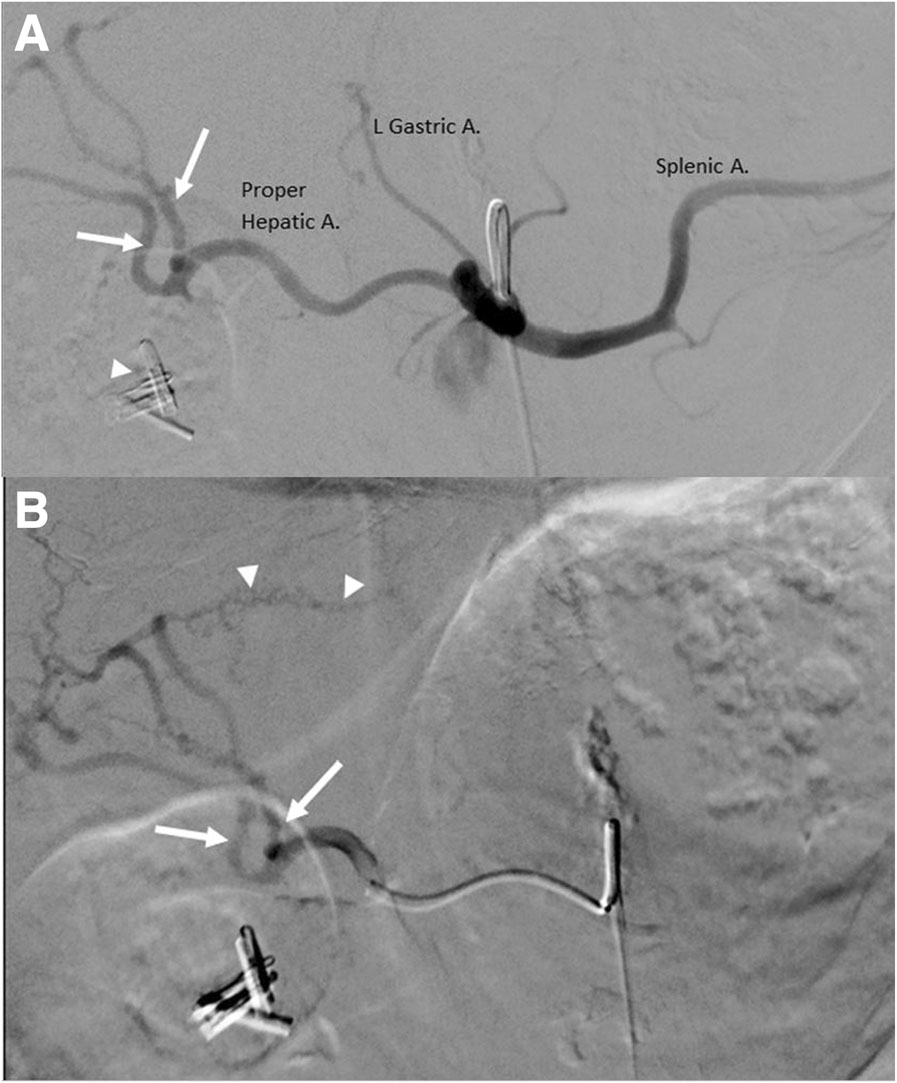
**a** Celiac axis angiogram demonstrating right and left hepatic arteries (arrows) arising from a proper hepatic artery (PHA) with no branching GDA. No blood flow is seen towards site of bleeding as identified by endoscopic clips (arrowhead). **b** PHA angiographic subselection demonstrating right and left hepatic arteries (white arrows), with LHA distribution opacified (arrowheads)

**Fig. 2:**
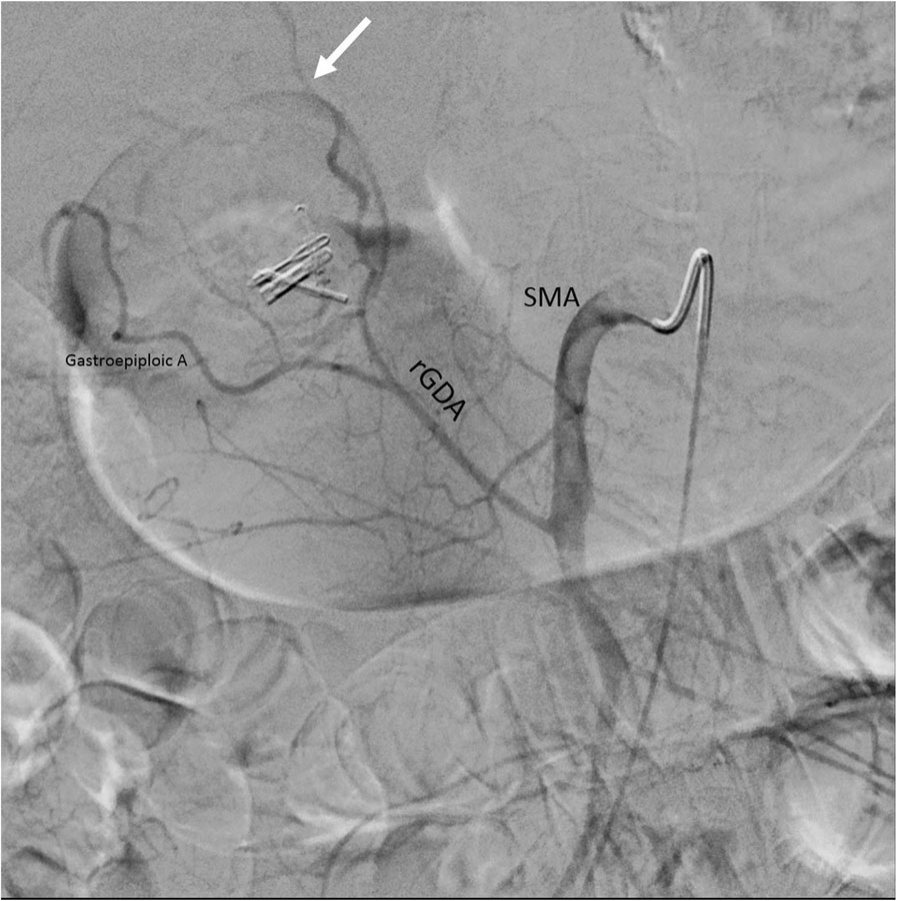
SMA angiogram demonstrating a replaced GDA (rGDA), which then continued towards the liver (arrow)

**Fig. 3:**
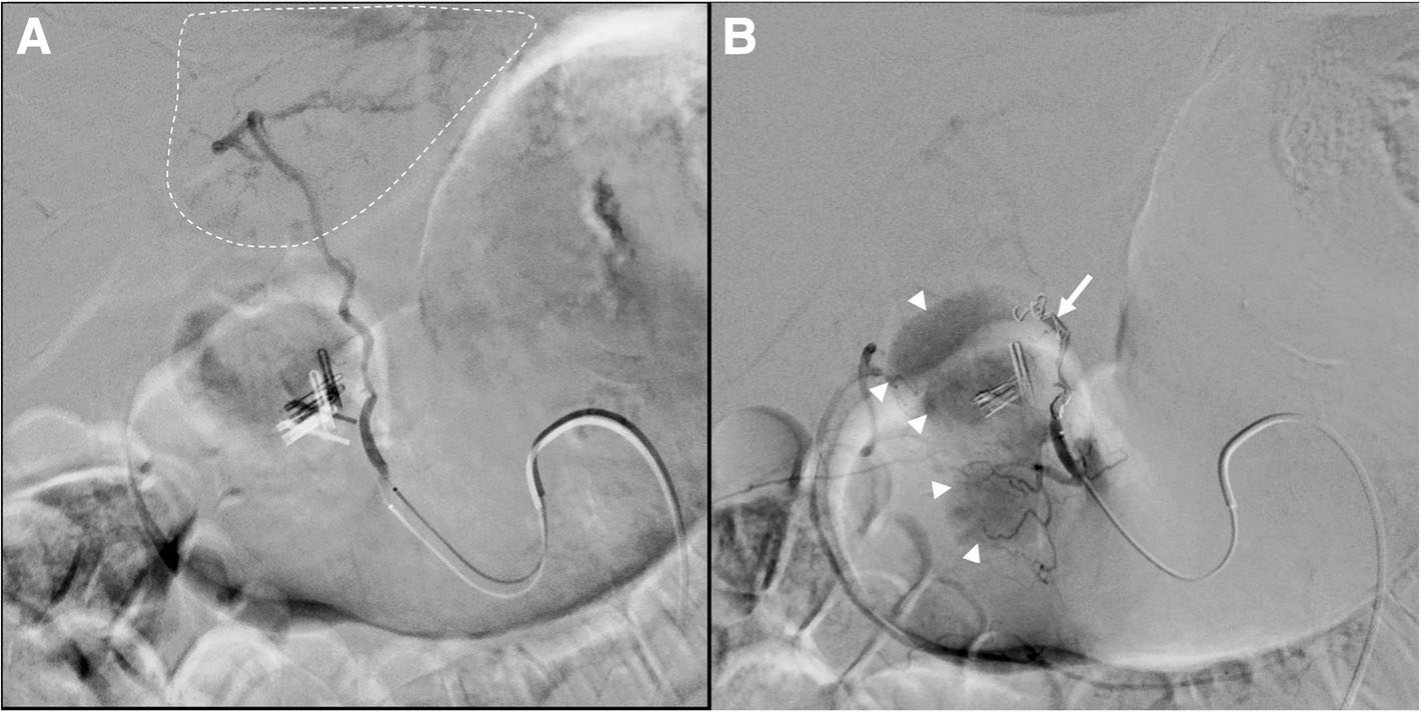
**a** GDA angiographic subselection demonstrating blood supply to the left lobe of the liver (dotted area), with *no opacification of the RHA*. **b** Active extravasation of contrast into the duodenal lumen in the region of the endoscopic clips was seen (arrowheads). There is decreased distal flow after initial coil placement (arrow). Additional coils were subsequently deployed

This 74-year-old male with recurrent peptic ulcer disease presented with acute onset weakness, hypotension, and melena. Following initiation of a rapid transfusion protocol, the patient was taken to the GI lab where arterial hemorrhage from an ulcer in the anterior duodenal bulb was identified. Upon upper endoscopy, epinephrine injection therapy and multiple clip application could not fully control bleeding. These endoscopic clips (in this patient with no prior abdominal surgery) later helped identify the site of bleeding on fluoroscopy. The patient was then brought to the IR suite for mesenteric angiography. Since the patient was borderline unstable, contrast enhanced CT was forgone. Based on the anatomic location of the visualized bleeding peptic ulcer, it was thought the arterial source of bleeding would be the GDA. However, upon catheterization of the celiac axis and subselection of the presumed CHA, angiography demonstrated *no native GDA* (Fig. 1a). Proper right and left hepatic arteries were identified (Fig. 1b). Superior mesenteric artery (SMA) angiogram demonstrated opacification of a replaced GDA (Fig. 2), which then supplied a large portion of the left lobe of the liver, *with no opacification of the right hepatic artery* (Fig. 3a). Active extravasation of contrast into the duodenal lumen in the region of the endoscopic clips was seen (Fig. 3b). The replaced GDA was successfully coil embolized, with post-coil embolization angiography demonstrating occlusion of the replaced GDA and patency of the gastroepiploic artery, which also arose from the GDA (seen in Fig. 2).

The patient’s symptoms of melena and hypotension resolved quickly after intervention. A liver function panel drawn the following day was within normal limits, however the liver panel drawn the following day on post-procedure day 2 demonstrated a mild transaminitis.

## Discussion

Within the field of interventional radiology, there has been a recent explosion of interest in trans-arterial therapies related to the liver and foregut organs including chemo- or radio-therapy, and various embolization techniques. The continual development of such interventions highlights the importance of understanding the variability in hepatic arterial anatomy.

This patient had a proper hepatic artery (with both right and left hepatic branches) arising from the expected location in the celiac axis. Thus, the artery arising from the SMA and giving off multiple branches to supply the duodenum could only be a replaced GDA. This vessel continued as an accessory LHA, likely supplying segments II and III. To the authors’ knowledge, this anatomic variation of the GDA with an accessory LHA is not described elsewhere.

The traditional classification of anatomic variation of the hepatic artery was introduced by Michels in 1955, where much of the known anatomic variability was identified by cadaveric dissection (Michels 1955). More recent meta-analyses and large surgical and radiological studies have offered a range of classification systems for the celiac trunk and CHA, and a review of this literature confirms how unique this variant may be (Noussios et al. 2017; Panagouli and Venieratos 2013; Hiatt et al. 1994; Song et al. 2010).

Panagouli et al. published a meta-analysis of celiac axis variation including 12,000 cases from 36 studies, and described a CHA replaced to the SMA in 1.13% of cases (Panagouli and Venieratos 2013). There was, however, no description of GDA replacement in the setting of a normal proper hepatic artery (PHA) from celiac trifurcation. Hiatt et al. described variant hepatic anatomy in 1000 patients who underwent hepatectomy, with a similar 1.5% rate of CHA replacement to the SMA, but again without mention of a PHA from the celiac axis (Hiatt et al. 1994). Likewise, Covey et al. described hepatic arterial anatomy in 600 patients evaluated by DSA, with 12 (2%) patients having CHA replaced to SMA, and 2 (0.3%) patients having PHA replaced to SMA with GDA coming directly from the aorta (Covey et al. 2002).

Most notably, Song et al. described 5002 patients by CT or DSA and described a replaced GDA in 1.1% of patients (specifically replaced to the SMA in 0.8% of patients), however there was no description of the GDA continuing into a hepatic arterial component (Song et al. 2010). Not surprisingly, this variant of an accessory LHA arising from the replaced GDA, with proper right and left hepatic arteries arising from a PHA is not readily explained by the anatomic model put forth by Song et al. They proposed a multi-level precursor anastomotic arterial pathway, part of which involves a primitive anastomosis of the left gastric artery, left hepatic artery, and celiac axis (the so-called “Lesser omental pathway”). This pathway would thereby explain the commonly-seen replacement of the LHA to the left gastric artery. This model, however, does not explain an accessory LHA arising from a replaced GDA.

An entirely replaced LHA on a GDA replaced to the SMA is described in 1 case report by Younan et al., however the patient described here is of added interest due to the incongruity with the previously published anatomical model described above (Younan et al. 2016). An entire LHA arising from the GDA conforms with Song’s anatomical model. In contrast, their “lesser omental pathway” does not communicate with the GDA and thus a new anastomotic channel would have to be conceived to explain the variant described here as this patient’s proper LHA maintains its normal anatomical location.

From a procedural perspective, this case demonstrates the importance of thorough interrogation of both the celiac and SMA axes during mesenteric angiogram. Upon initial nonvisualization of a GDA on celiac angiography, it may have been assumed that the GDA was in spasm or constricted secondary to epinephrine injection. However, secondary SMA angiography was critical in revealing the bleeding replaced GDA.

Upon discovery of this anatomic variant with terminal supply in the liver, it was considered intraoperatively that embolization of the GDA might result in ischemia of the downstream left lateral section of the liver. However, the patient had already received 6 units of packed red blood cells between the emergency department and the GI lab, and in the emergent setting presented here, and without known hepatic disease, this partial hepatic ischemia was determined to be an acceptable risk. Not surprisingly, this patient developed a mild transaminitis (AST = 107 u/L) on post operative day 2, which resolved 2 weeks later. The subsequent liver injury further corroborates the aberrant hepatic supply described above.

Interventional radiologists must have a comprehensive understanding of not only the standard hepatic and foregut arterial anatomy but also its variants in order to avoid complications, treatment failure, excessive radiation and contrast administration. The anatomical variant described here would have implications for interventional oncology in particular, where a treatment dose may need to be split during radiation lobectomy. Additionally, variations of hepatic arterial anatomy such as this are of particular importance for hepatobiliary surgeons. For example, variant arterial supply can complicate vascular dissection in organ procurement/transplantation or curative resection in surgical oncology (Younan et al. 2016).

## Conclusion

Described here is a rare hepatic arterial variant identified during mesenteric angiography for upper GI bleed. To the authors’ knowledge, continuation of a replaced GDA into an accessory left hepatic artery independent of the PHA is not described elsewhere. This variant also challenges a previously conceived anatomical model that attempts to explain observed CHA variation. Knowledge of the breadth of variant arterial anatomy has wide application in interventional radiology, particularly when mapping for focal intervention in the liver, and relevant prior imaging should be reviewed to avoid complication.

## Abbreviations

CHA: Common Hepatic Artery
GDA: Gastroduodenal Artery
LHA: Left Hepatic Artery
PHA: Proper Hepatic Artery
SMA: Superior Mesenteric Artery
